# Autofluorescence properties of balloon polymers used in medical applications

**DOI:** 10.1117/1.JBO.25.10.106004

**Published:** 2020-10-20

**Authors:** Huda Asfour, Jeremy Otridge, Robert Thomasian, Cinnamon Larson, Narine Sarvazyan

**Affiliations:** a The George Washington University, Department of Pharmacology and Physiology, Washington, DC, United States; b Nocturnal Product Development, LLC, Durham, North Carolina, United States

**Keywords:** autofluorescence, medical balloons, catheter design, hyperspectral imaging

## Abstract

**Significance:** For use in medical balloons and related clinical applications, polymers are usually designed for transparency under illumination with white-light sources. However, when illuminated with ultraviolet (UV) or blue light, most of these materials autofluoresce in the visible range, which can be a concern for modalities that rely on tissue autofluorescence for diagnostic or therapeutic purposes.

**Aim:** A search for published information on spectral properties of polymers that can be used for medical balloon manufacturing revealed a scarcity of published information on this subject. The aim of these studies was to address this gap.

**Approach:** The autofluorescence properties of polymers used in medical balloon manufacturing were examined for their suitability for hyperspectral imaging and related applications. Excitation-emission matrices of different balloon materials were acquired within the 320- to 620-nm spectral range. In parallel, autofluorescence profiles from the 420- to 620-nm range were extracted from hyperspectral datasets of the same samples illuminated with UV light. The list of tested polymers included polyurethanes, nylon, polyethylene terephthalate (PET), polyether block amide (PEBAX), vulcanized silicone, thermoplastic elastomers with and without talc, and cyclic olefin copolymers, known by their trade name TOPAS.

**Results**: Each type of polymer exhibited a specific pattern of autofluorescence. Polyurethanes, PET, and thermoplastic elastomers containing talc had the highest autofluorescence values, while sheets made of nylon, PEBAX, and TOPAS exhibited negligible autofluorescence. Hyperspectral imaging was used to illustrate how the choice of specific balloon material can impact the ability of principal component analysis to reveal the ablated cardiac tissue.

**Conclusions:** The data revealed significant differences between autofluorescence profiles of the polymers and pointed to the most promising balloon materials for clinical implementation of approaches that depend on tissue autofluorescence.

## 1 Introduction

Polymers provide great benefits such as durability, biocompatibility, disposability, and scalability. For use in medical balloons and related clinical applications, polymers are usually designed for transparency under illumination with white-light sources. However, when illuminated with ultraviolet (UV) or blue light, most of these materials autofluoresce in the visible range, which can be a concern for modalities that rely on tissue autofluorescence for diagnostic or therapeutic purposes.

One such modality is hyperspectral imaging based on tissue autofluorescence (Auf-HSI). This modality can be useful for a variety of clinical targets and has been widely explored for diagnostics of skin cancer,^1^ lymphoid tissue analysis,^2^ colon and gastric surgery,^3^ diabetes vasculopathies,^4^ and many other conditions.^5^^–^^7^ This is because many biological fluorophores have specific autofluorescence footprints in the 400- to 600-nm spectral range.^8^^,^^9^ Therefore, by assessing changes in tissue autofluorescence profiles within this critical range, one can monitor a wide range of pathophysiologic processes. Such processes include a buildup of the reduced form of nicotinamide adenine dinucleotide (NADH) during the ischemia,^10^^,^^11^ accumulation of collagen upon scar formation,^12^^,^^13^ age-related alternation in elastin-collagen content,^14^^,^^15^ buildup of lipofuscins in atherosclerotic plaques,^16^ and many other conditions. Thermal ablation, by causing acute changes in the tissue autofluorescence profile, is another example. Radiofrequency, cryo- or laser-based ablation irreversibly destroys cells causing an immediate drop in NADH autofluorescence.^17^^,^^18^ Observing this drop allows one to monitor the success of surgical ablation procedures performed both externally and internally, including endocardial surfaces of the heart.^19^^,^^20^ In the latter case, optically dense blood must be displaced from an optical path, either by influx of saline or via an inflatable balloon.^21^^,^^22^

Such balloons must exhibit minimal levels of fluorescence within optical ranges of biological fluorophores. Otherwise, they can diminish Auf-HSI’s ability to reveal the target tissue due to inherent autofluorescence of their material. Our search for published information on spectral properties of polymers that can be used for manufacturing of suitable medical balloons revealed only a few papers,^23^^–^^25^ but even those do not directly relate to this subject. Instead, manufacturer’s material sheets and peer-reviewed articles describe a myriad of mechanical and/or biologically-relevant properties, such as material hardness, tensile strength, degree of elongation, thermal stability, dielectric constant, hydrophobicity, photodegradation, and so on.^26^^–^^28^ As of today, to the best of our knowledge, very limited information, if any, is available on endogenous fluorescence of polymers from which one can manufacture transparent balloons for Auf-HSI or any other autofluorescence-based diagnostic modalities.^29^

Experiments detailed in this paper attempt to start filling the above-described knowledge gap. The selection of the polymers was based on their potential to create transparent balloons for medical applications. Balloon-equipped catheters are a staple of modern medicine and are used to treat numerous target organs. They are used to perform minimally invasive ablations,^30^^,^^31^ deliver drugs,^32^ position stents,^33^ and occlude or dilate vessels and cavities.^34^^,^^35^ In most of these procedures adding imaging capabilities to these catheters can be greatly beneficial.^36^ The list of materials examined in this study included polyurethanes,^37^^,^^38^ nylon,^39^ polyethylene terephthalate (PET),^23^ polyether block amide (PEBAX),^40^ thermoplastic elastomers (TPEs),^41^ vulcanized silicone,^42^ and cyclic olefin copolymers.^43^ All these materials can be used, in one form or another, to create transparent balloons for intravascular or intracavital use.

Medical balloons can be divided into three general types.^44^^,^^45^ Noncompliant, high durometer balloons are used for applications in which the balloon needs to expand to a specific diameter and exert high pressure to open a blockage or dilate the vessel or a cavity (compliance range 0% to 10%). They are typically made of PET, polyester, or nylon. Semicompliant balloons are commonly made of PEBAX or higher-durometer polyurethanes (compliance range 10% to 20%). They can create mid to high pressures but have more flexibility for easier delivery. Compliant balloons are typically made of mid to low durometer polyurethanes or silicone. These balloons are inflated by volume rather than pressure, enabling the balloon to conform to the anatomy of the surface being treated or imaged (compliance range 20% to 200% or more). Newer formulations, such as cyclic olefin copolymers^43^^,^^46^^,^^47^ or new combinations made of established polymers^38^^,^^41^^,^^48^ continue to expand the range of materials that can be used for manufacturing medical balloons.

For this study, we first acquired excitation-emission matrices (EEMs) from main types of commercially available polymer sheets using spectrophotometry-based approach. We then complemented these data with autofluorescence profiles of the same materials obtained from the Auf-HSI datasets. The data revealed significant differences between autofluorescence profiles of tested polymers and pointed to cyclic olefin copolymers (trade name “TOPAS”), as one of the most promising compliant balloon materials suitable for clinical implementation of Auf-HSI and other imaging approaches that depend on tissue autofluorescence. Lastly, the superiority of TOPAS sheets was confirmed by performing Auf-HSI of different cardiac tissues with and without this polymer in the optical path.

## 2 Methods

### 2.1 Polymer Sheets

To identify commercially available polymers most suitable for assessment of biological autofluorescence, we examined seven different classes of medical balloon materials. Specific samples of polymer sheets obtained from commercial sources are listed in Table 1.

### 2.2 Spectrofluorimetry

**Fig. 1:**
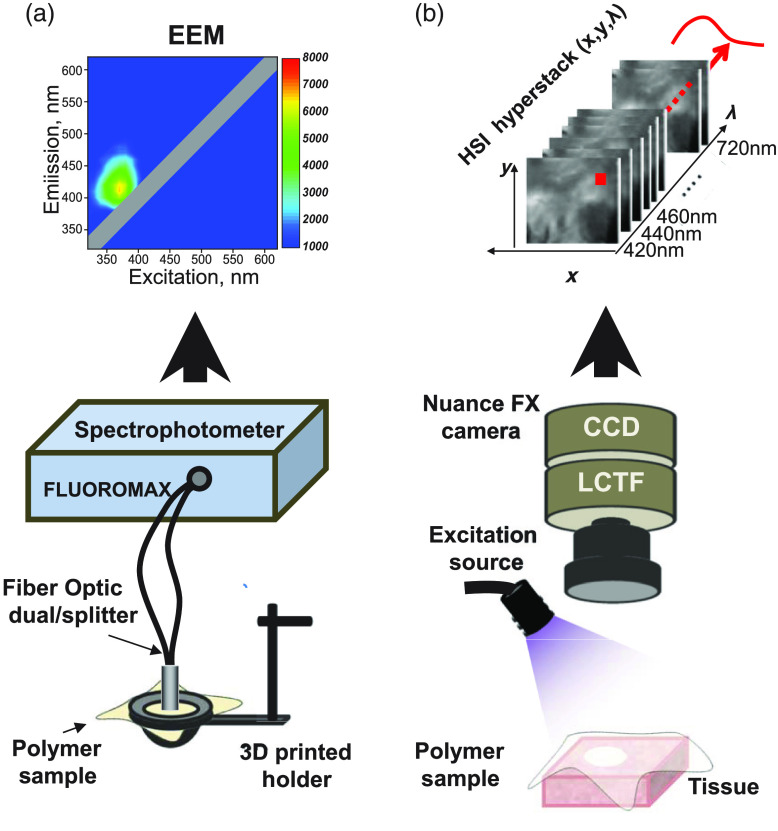
Imaging setup and equipment. (a). Bifurcated optical fiber was pressed against polymer surface stretched using a custom-made holder. EEMs were acquired using Fluoromax 3 spectrofluorimeter. (b) Nuance FX Hyperspectral system was used to collect a stack of 31 images from the 420- to 720-nm spectral range. The Mightex 365-nm LED was used to illuminate excised specimen with and without a polymer on their surface.

Excitation-emission matrix, commonly abbreviated as EEM, is a contour plot of excitation wavelength versus emission wavelength versus fluorescence intensity that provides a material’s spectral fingerprint. The EEM values found within a triangle formed by the diagonal reflectance line, the emission, and the excitation axes show sample autofluorescence. EEMs were collected via a bifurcated fiber-optic guide connected to a spectrofluorometer (HORIBA Jobin Yvon FluoroMax-3, Edison, New Jersey). The fiber was placed in direct contact with the flattened polymer mounted on a custom-made 3D-printed fixture sprayed with a non-fluorescent black coating [Fig. 1(a)]. The lens on the tip of fiberoptic guide had ∼2-mm diameter, while the dimensions of the mounted polymer surface were ∼2×2  cm. Emitted and reflected light were delivered back to the detector through the same bifurcated fiberoptic as shown in Fig. 1(a). EEM acquisition settings spanned from 320 to 720 nm. Since the 620- to 720-nm range did not have autofluorescence signals, EEM values shown in the figures are from the 320- to 620-nm range. Specular reflectance values were removed from the x and y coordinates of the same emission and excitation wavelengths on the EEM plots (i.e., intensity value of returning light acquired at the same wavelength as the sample was illuminated with). OriginPro2019 software was used to plot EEM color maps and other graphs.

### 2.3 EEM Variability

**Fig. 2:**
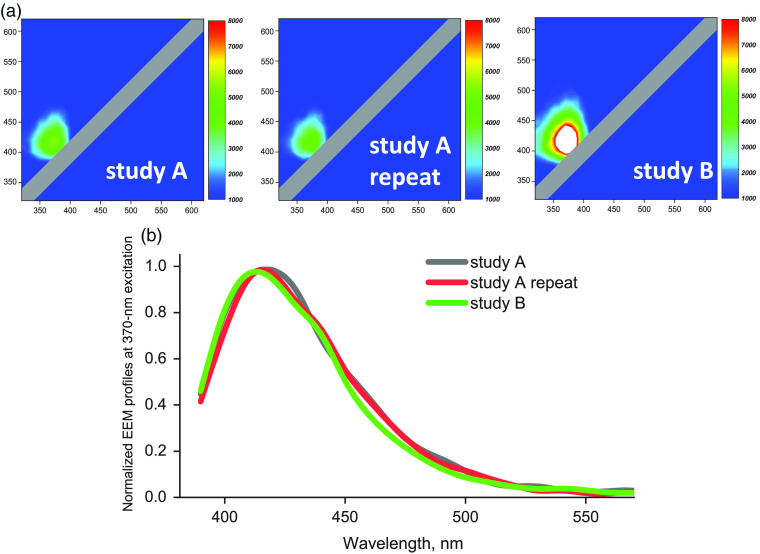
EEM variability. (a) Illustrative EEMs from high durometer polyurethane sheet from Nordson Medical (22003305AC, Shore 70D). All four EEMS use the same color scale. The upper two panels show two consecutive EEMs taken on the same day (labeled study A). The plot labeled as study B was taken during a different study day more than a month apart from study A and represents an extreme case of EEM variability. LUT scale on the right of each EEM show photon count. (b) Intensity profiles extracted from the above EEMs at the 370-nm excitation and normalized from 0 to 1.

When normalized to their peak fluorescence values, EEM profiles of each type of polymer were highly consistent across individual studies with variability <10% of the peak value. The absolute amplitude of autofluorescence signals, however, was much more variable. Such variability was minimal when successive EEMs were taken on the same day from the same sample without repositioning the probe. But, when EEMs were taken on different days using different samples from the same material sheet, the absolute values of the EEM varied. This is shown in Fig. 2 using EEM data for a polyurethane sheet from Nordson Medical (22003305AC, 70D). All EEMs shown in Fig. 2 were plotted using the same color scale. The upper two panels show two consecutive EEMs taken on the same day (labeled “study A”). The EEM plot labeled as “study B” was taken from a different piece of the same polymer sheet more than a month after study A and represents an extreme case of sample variability. The graph below displays the normalized autofluorescence profiles extracted from the EEM plots at the 370-nm value of the excitation axis. It illustrates that when normalized, the autofluorescence profiles are identical for all four EEMs shown above.

Similar patterns held true for all examined polymers. Higher variability of absolute EEM values can be explained by multiple factors, including minute differences in the stretch when mounting the polymer sample, small variations in the distance and the orientation angle between the material and the surface of the fiberoptic probe, and thickness inconsistency across different pieces of the same material sheet. Notably, many manufacturers report not a single value, but a range of thickness for each polymer (Table 1).

**Table 1:** Tested classes of polymers and their physical properties as reported by the manufacturer.

Polymer	Company	Serial number	Thickness (mil)	Material-shore hardness
Urethane very low durometer	Nordson Medical	20005500AC	1.15 to 1.65	Pellethane 2363 – 80A
Urethane low durometer	Nordson Medical	21003100AB	1.45 to 2.15	Pellethane 2363 – 90A
Urethane medium durometer	Nordson Medical	20003203AA	2.5 to 3.5	Pellethane 2363 – 55D
Urethane high durometer	Nordson Medical	22003305AC	2.0 to 2.6	Pellethane 2363 – 75D
PET	Nordson Medical	20032506AA	1.0 to 1.5	CAS25038-59-9 PET Homopolymer
Nylon	Nordson Medical	25006505BA	1.5 to 1.8	Grilamid L25
PEBAX	Nordson Medical	20032003AA	1.3 to 1.7	Pebax 70D
Topas E-140	TOPAS	F12-36-3	2.0, 4.0	—
Topas 8007	TOPAS	ETL 14-2-2	1.0	—
Vulcanized silicone	Vesta SIMATRIX	—	4.0	—
Urethane TSP1065	Polyzen	—	2.5	—
Urethane TSP1031	Polyzen	—	1.5	—
TPE NonTalc	Polyzen	—	N/A	—
TPE Talc	Polyzen	—	N/A	—

TPE – thermoplastic elastomer

### 2.4 Hyperspectral Imaging Protocols

Polymers were placed on either black cloth or on the top of animal tissue samples followed by illumination with 365-nm LED spotlight (Mightex, Pleasanton, California) oriented 5 to 8 cm from the sample surface [Fig. 1(b)]. Hyperstacks containing images from wavelengths ranging from 420 to 720 nm were collected using a Nuance FX hyperspectral imaging system (PerkinElmer, Waltham, Massachusetts) fitted with a Nikon AF Micro-Nikkor 60-mm f/2.8D lens. A 4×3  cm field of view was captured at 1392×1040  pixels, yielding a 30-μ/pixel spatial resolution. A region of interest-based selection, followed by the Nuance FX spectral unmixing algorithm, was used to extract lesion component images from each hyperstack as previously described in details.^19^ Alternatively, an automatic Nuance FX principal component analysis (PCA) algorithm was applied to reveal targets with different spectral profiles.

### 2.5 Tissue Sources and Ablation Procedures

Experiments were performed using freshly excised heart tissue from three different species: rats, pigs, and cows. Bovine and porcine hearts were obtained from a local abattoir or after surgical training at the Washington Institute of Surgical Education and Research. The explanted hearts were transported to the laboratory on ice within a 2- to 3-h window after the excision, followed by dissection to expose the relevant surfaces for ablation. RF energy was delivered with a non-irrigated ablation catheter (EP Technologies, Boston Scientific, Marlborough, Massachusetts). The 4-mm ablation tip was placed perpendicular to the endocardial surface, with ablation durations varying from 5 to 30 s and tip temperatures ranging between 50°C and 70°C. These settings created lesions similar in size to those placed during clinical RF ablation therapy, as detailed previously.^19^

### 2.6 Statistical Analysis

All values are presented as mean±SD, unless otherwise noted. Statistically different effects (p<0.05) between groups where determined according to one-way analysis of variance (ANOVA). It was followed by Tukey HSD post-hoc tests for pairwise comparisons. Evaluation of the correlation between EEM and HSI values was done by calculating the Pearson correlation coefficient. Representative EEMs and Auf-HSI images are shown across the figures.

## 3 Results

### 3.1 Presence of Polymer Impacts the Autofluorescence Profile of the Tissue to be Observed

**Fig. 3:**
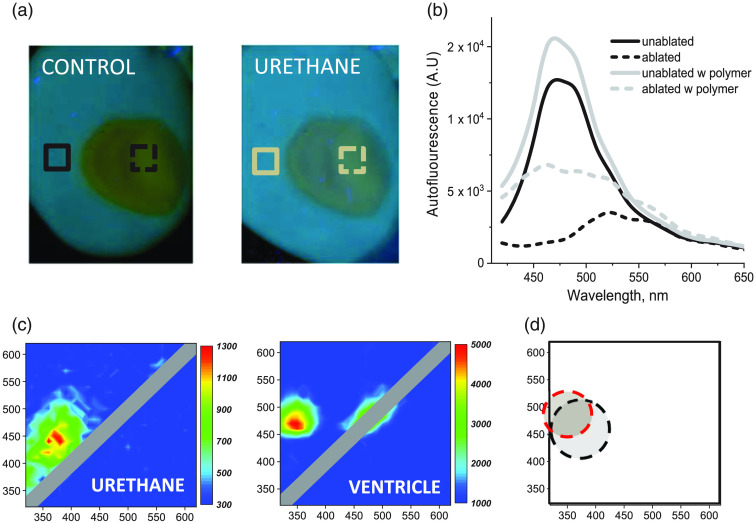
Effect of transparent polyurethane sheet on tissue autofluorescence signals. (a) Visual appearance of ablated rat ventricle illuminated with 365-nm UV light with and without a urethane sheet on its surface. (b) Representative spectra extracted from Auf-HSI hyperstacks using regions of interest shown in panel A. Presence of a polyurethane sheet (Nordson Medical, part 61) increases the overall intensity of autofluorescence and decreases the difference in spectral profiles between the lesion site (dotted line) and the rest of the tissue (solid lines). Black lines are from the experiment with no polymer, and gray lines are after the polymer was placed on top of the heart. (c) On the left is an EEM acquired from the polyurethane sheet used in panel B. On the right is an EEM from unablated rat ventricle showing broad NADH peak at ∼360  nm/450- to 460-nm excitation/emission wavelengths. (d) A cartoon to shows the spectral overlap between the main EEM peaks of the polymer (black dotted lines) and the unablated ventricle (red dotted line).

The most abundant biological fluorophores in cardiac tissues include NADH, collagen, and elastin. All of them have wide autofluorescence footprints in the 400- to 500-nm spectral range.^8^^,^^9^ Although most medical-grade balloons are fully transparent visually, they can autofluoresce in the same 400- to 500-nm spectral range impacting the autofluorescence profile of tissue to be imaged. The experiment shown in Fig. 3 displays such spectral interference. It shows a UV-illuminated, freshly excised rat heart with a large radiofrequency ablation lesion on its surface. When a sheet of clear polyurethane is placed on the specimen, a smaller contrast is observed between the native and ablated tissue [Fig. 3(a)]. This qualitative visual observation can be quantified by comparing the differences in the spectral profiles of ablated and native tissue in the presence or absence of the polymer [Fig. 3(b)]. The excitation-emission plots of the polyurethane sheet and heart ventricular surface before ablation confirm that their autofluorescence profiles largely overlap [Figs. 3(c) and 3(d)]. Importantly, in this illustrative experiment, spectral differences between ablated and unablated tissue were very pronounced while image acquisition was done *ex vivo*, using a high numerical aperture lens, plenty of illuminating light, and a long acquisition interval. All of which yielded spectra and images with high spectral and spatial resolution. In the case of smaller differences between the spectra of interest and/or when more challenging acquisition settings are to be used (i.e., during catheter-based signal acquisition), the interference from balloon material autofluorescence can potentially interfere with spectral unmixing that reveals the desired targets. The illustrative experiment shown in Fig. 3 therefore highlights the need to identify materials more suitable for making compliant balloons for Auf-HSI catheters and other imaging or diagnostic technologies that rely on tissue autofluorescence.

### 3.2 Different Polyurethanes

**Fig. 4:**
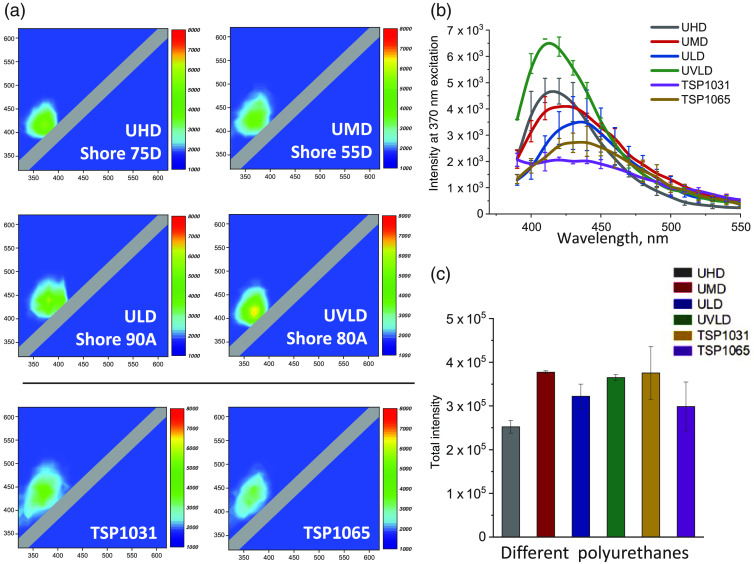
EEMs of different polyurethane sheets (a) Representative EEMs for polyurethane sheets with different durometer values. The upper four EEMs are for polyurethane samples from Nordson Medical, and the bottom two EEMs are polyurethane sheets from Polyzen (details in Table 1). (b) Autofluorescence profiles of the examined polyurethane polymers illuminated with 370 nm. (c) The total amount of autofluorescence from the entire 320- to 620-nm range.

One of the most commons classes of balloon materials is polyurethane.^28^ Polyurethanes contain copolymer blocks of high molecular-weight polyols linked together by a urethane group. They exhibit excellent biocompatibility and have the versatility of being rigid, semirigid, or flexible. This enables creation of transparent polyurethane sheets with different degrees of hardness based on material durometer values. Polyurethanes of different durometers have slightly different composition that is why polyurethane sheets from Nordson Medical with four different levels of hardness were examined (described in material sheets as very low, low, medium, and high hardness with shore values 80A, 90A, 55D, and 75D, respectively). EEMs of all tested polyurethane samples, regardless of their hardness values, revealed significant autofluorescence with wide excitation/emission peaks of ∼360 to 370 nm and ∼420 to 430 nm, respectively [Fig. 4(a)]. The average peak amplitude of the polyurethane EEMs of different degrees of hardness did not correlate with their thickness or durometer values (Table 1 and Fig. 4). Polyurethane sheets from another manufacturer (Polyzen TSP1031 and TSP1065) have shown a similar autofluorescence footprint [Fig. 4(a), bottom row]. The graph in Fig. 4(b) shows the mean autofluorescence profiles of the six polyurethane sheets extracted from the 370-nm excitation coordinate of their respective EEMs. Figure 4(c) compares the total amount of autofluorescence from the entire 320- to 620-nm range (calculated as described in Ref. 49). Despite expected minor differences in spectral profiles from individual polyurethanes, the total amount of fluorescence and the overall spectral footprints were quite similar. Together, these data confirmed that polyurethanes are poorly suitable for applications that rely on tissue autofluorescence since most common biological fluorophores, such as NADH, collagen, and elastin, autofluoresce in the same spectral range.

### 3.3 Comparing Spectral Properties of Different Polymer Classes

**Fig. 5:**
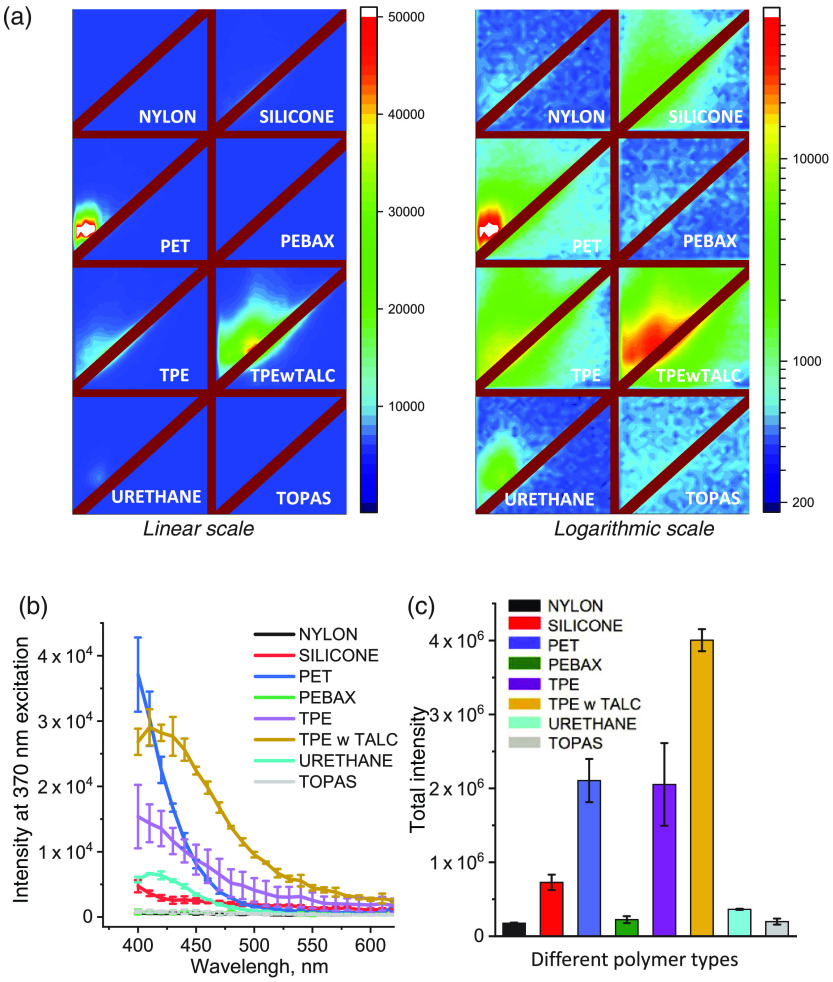
EEMs of different polymer classes. (a) Representative EEMs for different polymer classes. The panel on the left shows EEM values using a linear scale, while the panel on the right shows the same data using a logarithmic scale. (b) The mean intensity profiles of returning light along the 370-nm excitation line for each type of tested polymers. (c) The total amount of autofluorescence from the entire 320- to 620-nm range calculated for each type of tested polymer.

Next, we examined other classes of commercially available balloon materials. Figure 5 shows representative EEM patterns from each of the seven classes tested. The panel on the left shows EEM values using a linear scale, while the panel on the right show the same data using a logarithmic scale. To compare the EEMs quantitively, we extracted the intensity profiles of returning light along the 370-nm excitation line [Fig. 5(b)]. Notably, 370 nm was not necessarily the excitation wavelength that yielded the maximum fluorescence for all polymers. For example, looking at the EEM of PET, that wavelength will be closer to 320 nm or even lower. Thus, graphs shown in Fig. 5(b) are just first-order estimates of the sample autofluorescence. We also calculated the total autofluorescence value for each type of polymer by summing the intensity values from an entire 320- to 620-nm range. Therefore, when comparing any two polymers, a combination of color adjusted EEMs from Fig. 5(a) and graphs shown in Figs. 5(b) and 5(c) should be used.

For example, samples of TPE from Polyzen exhibited moderate levels of autofluorescence when illuminated with 370-nm light. Yet, when combined with talc (a clay mineral composed of hydrated magnesium silicate), its fluorescent pattern shifted, and the material became highly fluorescent when illuminated with 370-nm light. Polymer sheets made of nylon, silicone, and PEBAX had the lowest fluorescence values.

Another promising material was TOPAS, a relatively new type of polymer made of cyclic olefin copolymers.^47^ Multiple beneficial properties of TOPAS polymers are noted in their material sheets from their manufacturer (TOPAS Advanced Polymers). They include high compliance, superior chemical resistance, low water absorption, high UV transparency, and exceptional electrical insulation performance. All four different samples of TOPAS sheets that were tested had negligible autofluorescence. These included two different lots of 1-mil-thick sheets of TOPAS 8007 and two different sheets of TOPAS E140, one 2 mil, and another 4-mil-thick.

Statistical interpretation of EEM data can be done based on either total fluorescence values or individual wavelength of interest. The latter will depend on a particular set of filters or spectral acquisition settings that are user dependent. In the Supplementary Material, we included the examples of ANOVA analysis with post-hoc Tukey for data shown in Figs. 4 and 5. It confirms that total fluorescence of PET, TPE and TPE with talc significantly differ from that of nylon, silicone, PEBAX, and TOPAS. The latter four can be classified as low fluorescence compounds. Notably, summing up fluorescence values across the entire excitation/emission range, diminishes the spectral differences between the individual polymers. More useful information can be gained by comparing extracted fluorescence profiles for polymers of interest at specific wavelengths. For example, when excited at 370 nm, ultra-low durometer polyurethane is about three times more fluorescent compared to TOPAS and the difference between the two is highly significant across the entire 400- to 500-nm range. Similarly, while TPE and PET do not differ as far as their total fluorescence values, fluorescence intensity of PET is significantly higher than that of TPE within 400- to 440-nm range.

### 3.4 Validation of EEM Data by Auf-HSI Imaging

**Fig. 6:**
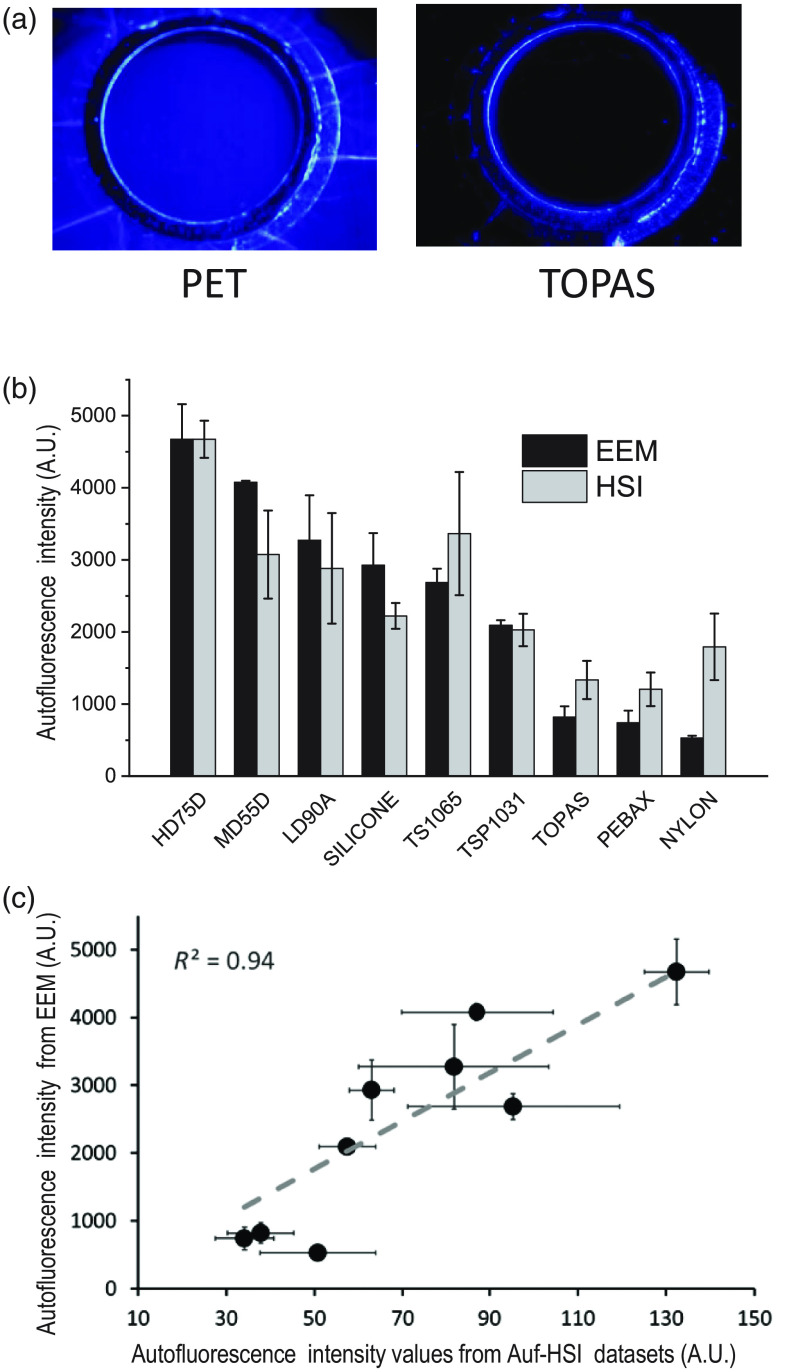
Validation of EEM data by Auf-HSI imaging. (a) Visual appearance of polymer sheets under 365-nm Mightex LED illumination when mounted into a round holder. Examples shown are polymer materials with very high- and very low-autofluorescence levels. (b) Relative values of autofluorescence intensity at the 370/420-nm excitation/emission point of EEM (black bars) versus corresponding intensity values from Auf-HSI of selected polymers samples. The latter values were obtained by placing pieces of the polymers on a black cloth and illuminating them with 365-nm Mightex LED from above (gray bars). The raw Auf-HSI values were multiplied by a constant to start from the same value as EEM of the high durometer urethane (HD75, first column). (c) Correlation plot between the arbitrary intensity values at the 370/420-nm EEM excitation/emission point and the corresponding intensity values from Auf-HSI profiles. Due to a much larger dynamic range of photomultiplier tube, polymers with the highest fluorescence values (PET and TPE with TALC) were excluded from the correlation analysis.

To validate EEM-based comparison between the polymers we conducted additional experiments using a hyperspectral imaging setup. The polymer pieces were either mounted into a round holder [Fig. 6(a)] or placed on a black cloth and illuminated using 365-nm Mightex LED. From a hyperstack of 31 images within the 420- to 620-nm range, the spectra from individual ROIs were extracted and contrasted with the corresponding EEM values along the 370-nm excitation line of the respective EEM. The graph in Fig. 6(b) compares the relative intensity values at the 370/420-nm excitation/emission point across nine different polymers obtained by either EEM (black bars) or Auf-HSI measurements (gray bars). Fig. 6(c) shows the correlation plot between these values (note: due to an inherently much larger dynamic range of the photomultiplier tube versus the CCD camera, the two polymers with the highest fluorescence values—PET and TPE with TALC were excluded from correlation analysis).

### 3.5 Confirmation of TOPAS Utility for Imaging of Biological Samples

**Fig. 7:**
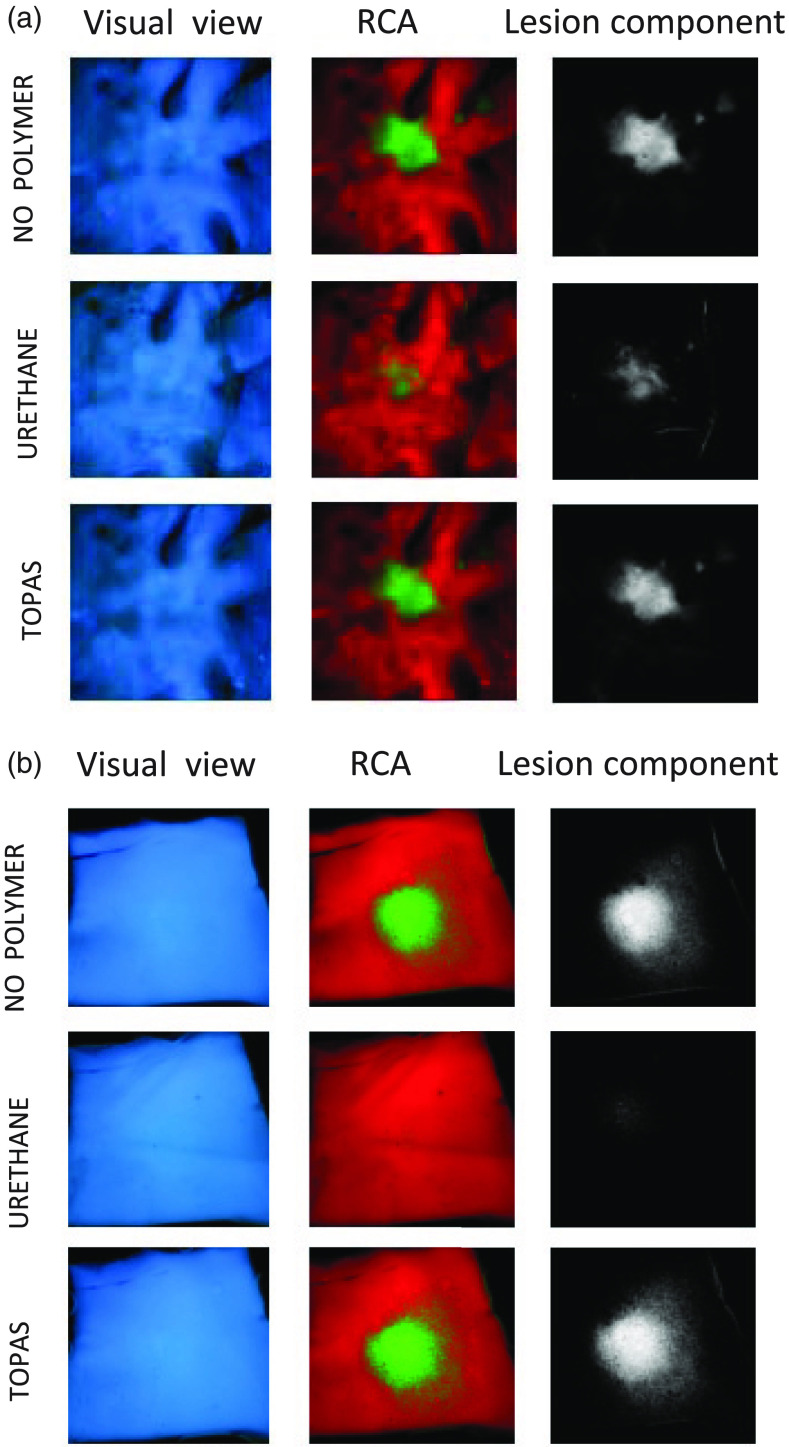
Impact of polymer material on spectral unmixing of target tissue. (a) Piece of excised porcine left atrium with radiofrequency ablation lesion in the center. The left column shows the visual appearance of the tissue under 365-nm Mightex LED illumination. Overabundance of endocardial collagen obscures damage to the muscle below when observed visually. Use of Auf-HSI reveals the lesion by applying PCA. The central column shows composite images with ablated tissue in green, unablated in red. The right column shows lesion component in grayscale. The three rows represent images from the sample without any polymer on its surface, with polyurethane (Nordson Medical, 20003203AA, medium durometer 55D) and with TOPAS (#E140, 2 ml). (b) Similar experiment using a piece of excised porcine aorta.

**Fig. 8:**
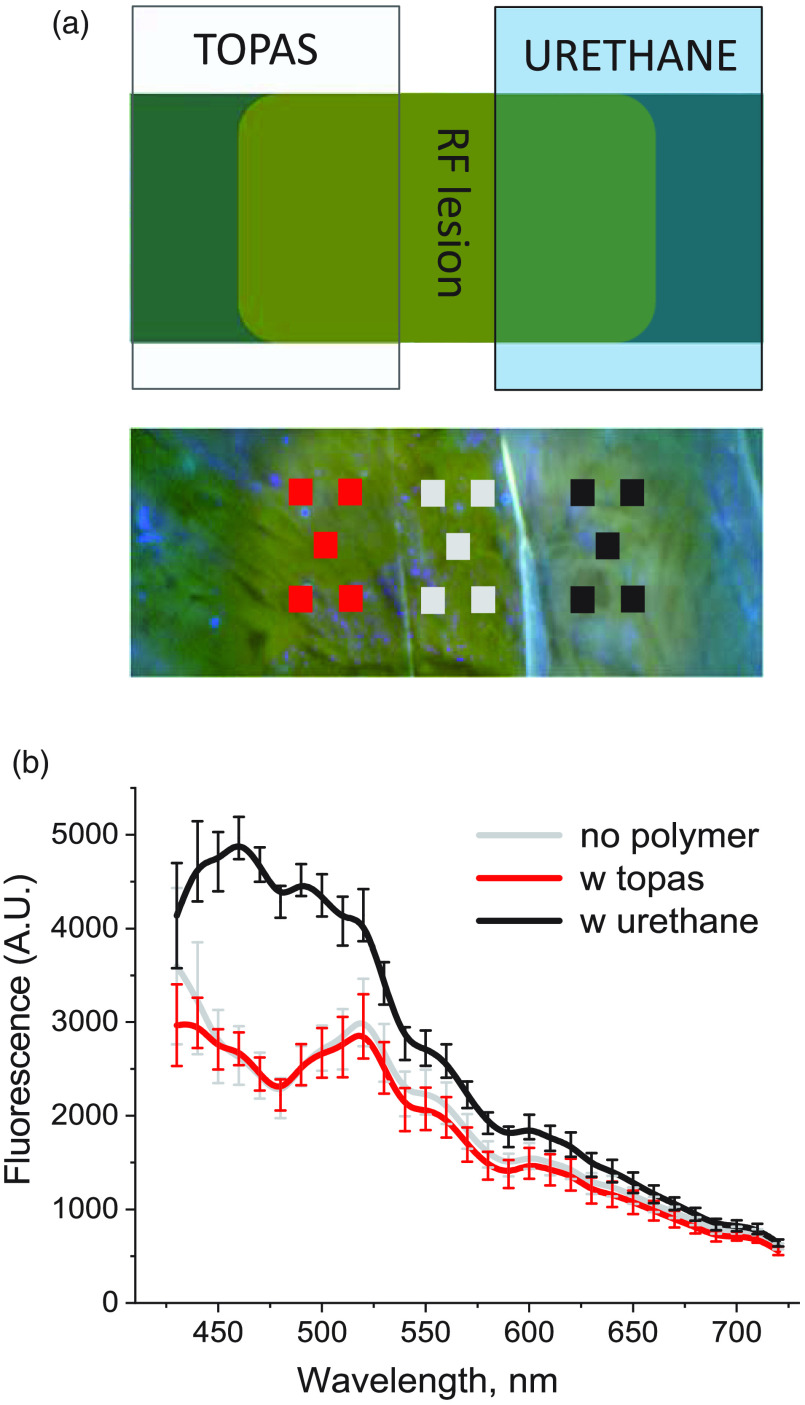
Side-by-side comparison of two different polymer materials and their effect on spectral unmixing of target tissue. (a) Two strips of polymers were placed on the top of a radiofrequency lesion made on a wedge of ventricular muscle from a cow. Auf-HSI was done under UV illumination as shown in Fig. 1 (b). The spectral profiles where extracted from multiple regions of interest placed on the lesion without polymer (gray), with TOPAS (red), and with polyurethane (black). (b) Autofluorescence profiles of the lesion site in the absence (gray trace) and presence of the TOPAS (red trace) or polyurethane (black trace). Profile values in the presence of TOPAS did not statistically differ from the lesion values across the entire tested range. Profile values in the presence of polyurethane were significantly different from that of the lesion across the 440- to 630-nm range.

Our next set of studies compared the usefulness of selected polymers as future balloon materials for diagnostic methods that rely on autofluorescence of biological samples. Specifically, we acquired hyperspectral datasets from freshly excised and ablated cardiac tissues with and without polyurethane or TOPAS sheets in the optical path. Figure 7 shows two types of tissue: an excised porcine left atrium and a piece of porcine aorta, which were ablated using a standard radiofrequency catheter. When a sheet of TOPAS was placed on ablated tissue, it did not have any impact on the ability of PCA to reveal the lesion. In contrast, use of a polyurethane sheet either diminished [Fig. 7(a)] or completely hindered [Fig. 7(b)] identification of ablated tissue. Figure 8(a) shows a closeup of ablated ventricular muscle with strips of TOPAS and polyurethane on top of it. The spectra acquired from the ablation site in the absence and the presence of the polymers show added fluorescent intensity from polyurethane, particularly in the 420- to 500-nm range, and the absence of any additional fluorescence from the TOPAS sheet [Fig. 8(b)].

**Fig. 9:**
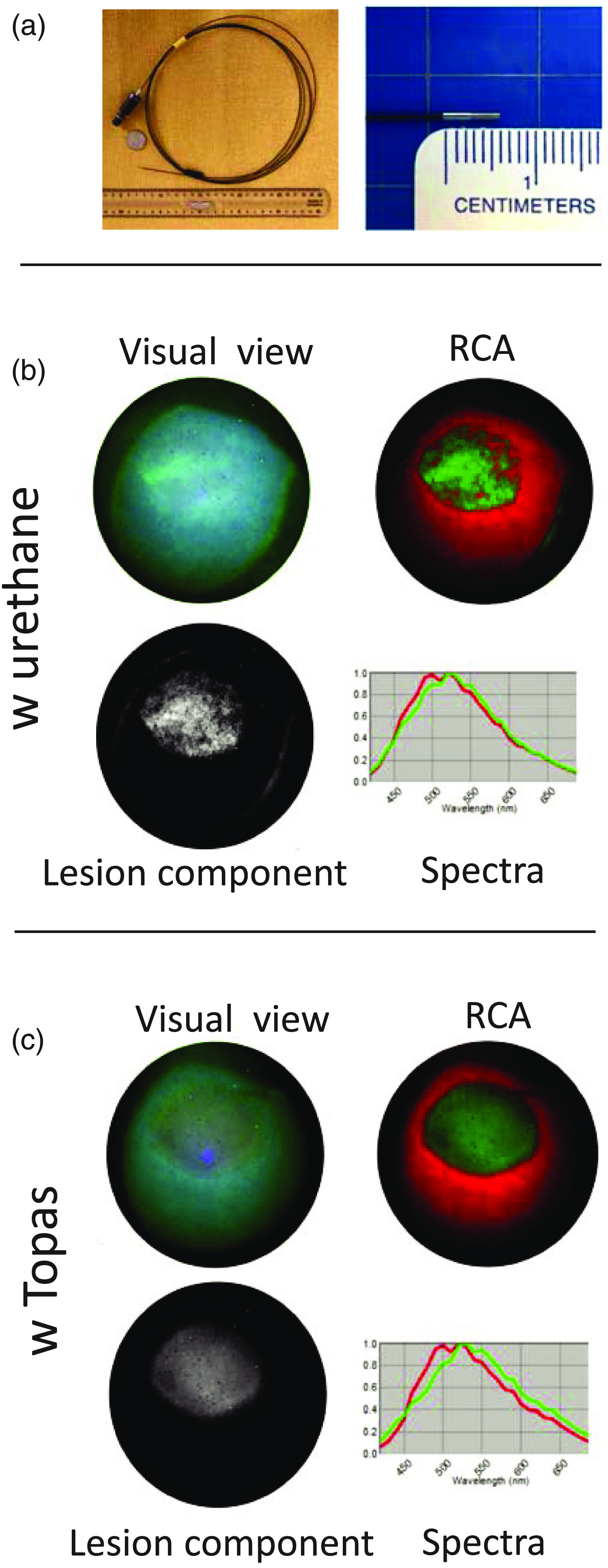
Testing balloon materials using image guide from percutaneous catheter. (a) Insertable imaging guide from percutaneous hyperspectral imaging catheter (Details in Swift et al.^22^). The image guides were assembled by Zibra Corporation, using Fujikura FIGH-17-600G coherent imaging fiber which comprises of 17,000 individual fibers and together with the attached lens has an outer diameter of 800  μ. (b) The upper left panel shows RGB image of an unprocessed hyperstack of ablated rat heart with polyurethane sheet on its surface. The hyperstack was acquired using the percutaneous catheter assembly connected to the Nuance FX HSI system with illumination guide connected to the 355-nm argon laser. The top right image shows composite image with ablated tissue in green, unablated in red. The bottom left image shows lesion component using grayscale. The graph on the right shows the difference in normalized spectral profiles of red and green components. The outcomes of unmixing using the same settings as in panel B above, but when tissue was imaged in the presence of TOPAS (#E140, 2 ml).

Lastly, we acquired hyperspectral datasets from ablated atrial tissue using a multimode imaging guide from a percutaneous catheter [Fig. 9(a)]. The image guide yields images with much lower spatial resolution and with less photon count since its overall diameter is <1  mm (compared to the Nikon AF Micro- Nikkor 60-mm f/2.8D lens attached to the Nuance FX camera used in experiments shown in Figs. 2, 6, and 7). This can make identification of target spectra that differ only slightly, particularly challenging. Indeed, inclusion of urethane in the optical path of the imaging guide, confounds spectral unmixing of the lesion site versus unablated tissue [Fig. 9(b)]. When replaced with TOPAS, the lesion appearance become nearly identical to the one without the polymer [Fig. 9(c)].

## 4 Discussion

Our goal was to survey the autofluorescence properties of commercially available polymers suitable for the fabrication of transparent balloons in medical applications. Admittedly, the list of available polymers continues to expand, and our selection did not encompass all materials on the market. Yet, we believe that the collected data includes the main classes of available polymers, helping to fill the existing gap on this subject in the published literature. The EEM data shown in the figures can be useful to many in the fields of optics and medical instrumentation.

Notably, we did not limit our experiments to high compliance balloon materials which are the most suitable for imaging applications such as Auf-HSI. Instead, we included non-compliant polymers such as PET or high-durometer polyurethane. This is because the list of so-called combination or multimodality catheters has been expanding.^50^^–^^52^ These newer devices aim to achieve several therapeutic goals at once by combining different diagnostic modalities. For example, in addition to simply opening the vessel, the proposed device can also image the wall surface or perform an ablation procedure. For these multimodal approaches, more properties of balloon material must be taken into account, including the foreseen need for polymers with intrinsically low-autofluorescence levels.

An increasing number of pre-clinical studies and an expanding range of clinical applications are based on spectral analysis of tissue autofluorescence.^53^ The presented data furthers these efforts by characterizing the fluorescence of the balloons required for visualization in diagnostic and surgical purposes. Beyond cardiac applications, Auf-HSI can be applied to treating conditions of the colon,^54^^,^^55^ for the diagnosis of atherosclerosis and neoplasia,^56^ to track the progress/health of tissue transplants,^57^ to provide live guidance for surgeons,^58^ and contribute toward developing and tracking cancer-specific target probes.^59^

The nylon and urethane polymers we studied are commonly used in medical devices and therefore have more information about their physical and spectral properties^25^^,^^60^ than other materials considered in our studies. Our findings contribute to the knowledge about all of these polymers, but in particular address the larger information gaps about Pebax, PET, TPEs, and TOPAS.^23^^,^^40^^,^^61^ The data point to TOPAS polymers as the least autofluorescent material making it most suitable for manufacturing compliant medical balloons. TOPAS is advertised as “glass-clear plastic” and its advanced properties include high glass transition temperatures up to 170°C, pronounced chemical resistance, low birefringence and very low water absorption (<0.01%). In addition, the material’s low dielectric properties provide strong electrical insulation performance. The material can withstand gamma or e-beam sterilization and can be injection-molded or extruded into film, sheet, tubing, or other components using conventional processing equipment. Our studies add the near absence of UV-elicited autofluorescence to the list of TOPAS’s beneficial properties.

Surprisingly though, out of the four TOPAS sheets tested, the thinnest one was the most autofluorescent. While we do not have specific information as to why, this unexpected finding points to the fact that to improve certain properties of the polymers, such as durability, manufacturers include additional components and/or modify polymer chemical structure to suit their goals. This was further highlighted by a side-by-side comparison of TPE with and without talc, a hydrated magnesium silicate (Fig. 5). Talc is commonly used to reinforce and improve dimensional stability of plastics and improve their processing abilities and mechanical properties. Inclusion of talc increased fluorescence levels manifold, making TPE with talc the most autofluorescent material on our list.

Today, a wide variety of coatings can be added to the surface of a medical balloon to enhance or change its properties to meet customer’s requirements. This includes coatings that makes the material more hydrophilic or hydrophobic, abrasion- and puncture-resistant coatings, conductive coatings, antithrombogenic coatings, and drug-release or reflective coatings. Based on data presented in this paper, our recommendation will be to test relevant spectral properties of any chosen polymer before proceeding with balloon design and implementation.

## Code, Data, and Materials Availability

No proprietary or newly created materials were used in these studies. The commercial sources of polymers are listed in Table 1. EEM data for specific polymer(s) are available upon email request to the corresponding author.
