# Supplementary material for: Autofluorescence properties of balloon polymers used in medical applications
Source: J Biomed Opt. 2020 Oct 20;25(10):106004. doi: 10.1117/1.JBO.25.10.106004 (PMC7575097; doi:10.1117/1.JBO.25.10.106004)
Supplement: Supplementary file 1 [file JBO_025_106004_SD001.pdf]

Tukey post-hoc test

credit to astatsa.com

Fig.4

Total FL

	UMD	ULD	UVLD	TSP1031	TSP1065
UHD	** $p<0.01$	* $p<0.05$	** $p<0.01$	** $p<0.01$	insignificant
UMD		insignificant	insignificant	insignificant	** $p<0.01$
ULD			insignificant	insignificant	insignificant
UVLD				insignificant	* $p<0.05$
TSP1031					insignificant

excitation 370nm **emission 420nm**

	UMD	ULD	UVLD	TSP1031	TSP1065
UHD	insignificant	* $p<0.05$	** $p<0.01$	** $p<0.01$	** $p<0.01$
UMD		insignificant	** $p<0.01$	** $p<0.01$	* $p<0.05$
ULD			** $p<0.01$	insignificant	insignificant
UVLD				** $p<0.01$	** $p<0.01$
TSP1031					insignificant

Fig.5

Total FL

	silicone	PET	PEBAX	TPE	TPE w talc	urethane	TOPAS
nylon	insignificant	** $p<0.01$	insignificant	** $p<0.01$	** $p<0.01$	insignificant	insignificant
silicone		** $p<0.01$	insignificant	** $p<0.01$	** $p<0.01$	insignificant	insignificant
PET			** $p<0.01$	insignificant	** $p<0.01$	** $p<0.01$	** $p<0.01$
PEBAX				** $p<0.01$	** $p<0.01$	insignificant	insignificant
TPE					** $p<0.01$	** $p<0.01$	** $p<0.01$
TPE w talc						** $p<0.01$	** $p<0.01$
urethane							insignificant

excitation 370nm **emission 420nm**

	silicone	pet	pebax	TPE	TPE w talc	urethane	topas
nylon	insignificant	** $p<0.01$	insignificant	** $p<0.01$	** $p<0.01$	** $p<0.01$	insignificant
silicone		** $p<0.01$	insignificant	** $p<0.01$	** $p<0.01$	insignificant	insignificant
PET			** $p<0.01$	** $p<0.01$	* $p<0.05$	** $p<0.01$	** $p<0.01$
PEBAX				** $p<0.01$	** $p<0.01$	** $p<0.01$	insignificant
TPE					** $p<0.01$	** $p<0.01$	** $p<0.01$
TPE w talc						** $p<0.01$	** $p<0.01$
urethane							** $p<0.01$
